# Supplementary figures and images for: Domain-specific transfer learning in the automated scoring of tumor-stroma ratio from histopathological images of colorectal cancer
Source: PLoS One. 2023 May 26;18(5):e0286270. doi: 10.1371/journal.pone.0286270 (PMC10218718; doi:10.1371/journal.pone.0286270)

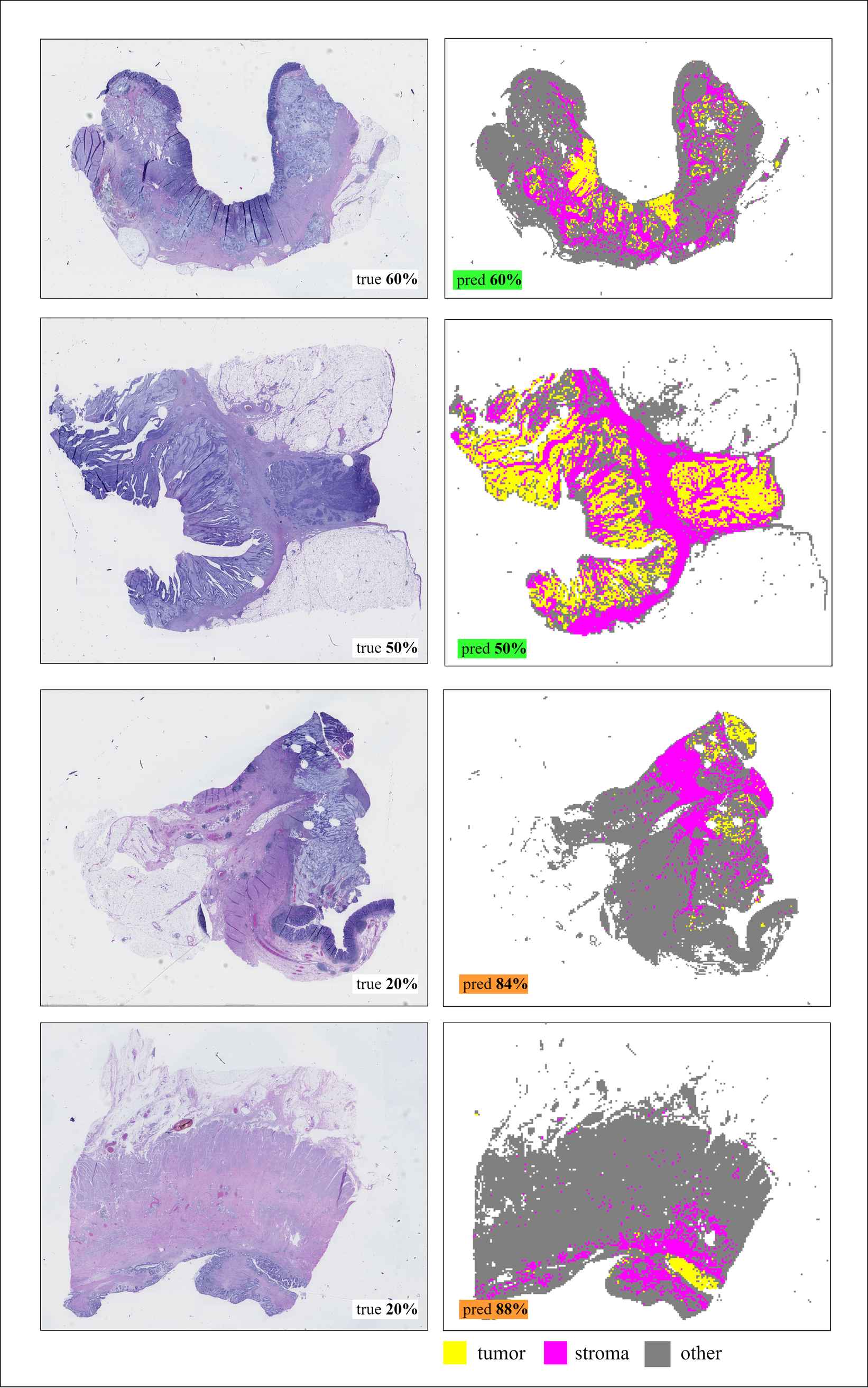

Supplement: S1 Fig — Examples of TSR prediction on four WSIs. (TIF) [file pone.0286270.s002.tif]
